# Satisfactory levels of functionality and quality of life in caregivers of patients with bipolar disorder: is this possible?

**DOI:** 10.47626/1516-4446-2024-3866

**Published:** 2025-01-22

**Authors:** Mariana Troesch, Sarah Prates, Stella Sarmento, Thiago Cerqueira-Silva, Gabriela Léda-Rêgo, Caroline Dallalana, Juliana S. Casqueiro, Paula Studart-Bottó, Ângela Miranda-Scippa

**Affiliations:** 1 Centro de Estudos dos Transtornos de Humor e Ansiedade, Universidade Federal da Bahia (UFBA), Salvador, BA, Brazil; 2 Programa de Pós-Graduação em Medicina e Saúde, UFBA, Salvador, BA, Brazil; 3 Fundação Oswaldo Cruz, Salvador, BA, Brazil; 4 Departamento de Neurociências e Saúde Mental, Faculdade de Medicina, UFBA, Salvador, BA, Brazil

**Keywords:** Functionality, quality of life, caregiver, bipolar disorder

## Abstract

**Objective:**

Functionality and quality of life levels were measured in caregivers of patients with bipolar disorder, investigating the associations between them and with clinical and sociodemographic data.

**Method:**

A cross-sectional study was conducted between 2020 and 2022 with caregivers of patients with bipolar disorder treated in a university hospital’s outpatient clinic. The following instruments were applied: a clinical and sociodemographic questionnaire, the World Health Organization Disability Assessment Schedule 2.0, the 36-Item Short Form Health Survey, the Mini International Neuropsychiatric Interview Plus, and the Structured Clinical Interview for DSM-IV Axis II Personality Disorders.

**Results:**

The median World Health Organization Disability Assessment Schedule 2.0 score was < 20, and the median score on the 36-Item Short Form Health Survey domains was > 60. Caregivers with greater functional impairment had lower quality of life. Being younger, being a parent, having non-psychiatric clinical conditions, and having mental disorders were associated with lower quality of life and functionality.

**Conclusion:**

These results indicate less functional impairment and better perceived quality of life than what has been reported in the literature, demonstrating that there is a subgroup of caregivers who perform their role without significant compromise to these health aspects. Exploring other factors associated with functionality and quality of life, such as resilience, psychological support, etc., may allow for more targeted public health interventions, optimizing comprehensive bidirectional care for patients and caregivers.

## Introduction

Bipolar disorder (BD), a severe, chronic, and recurrent illness, is estimated to affect 2.4% of the world population.^1^ Each episode is a stressful event for caregivers of individuals with this disorder, and the high level of distress often negatively affects their physical and mental health.^2^ The literature reports that depressive symptoms, feelings of burden, and seeking health care services are associated with caregivers of individuals with BD.^3^,^4^ Indeed, a bidirectional cycle is believed to exist in which the repercussions of BD on caregivers interfere with the quality and capacity of care.

A recent study showed that 67.7% of caregivers considered their quality of life (QoL) to be moderately or extremely affected by their patient’s BD.^5^ Various aspects of the caregivers’ lives are impaired, including mental health, physical health, and social activities.^6^ Functionality is a broad and complex concept, described as the individual’s ability to perform tasks related to daily life, such as work activities, recreational activities, living autonomously, and engaging in mutually fulfilling relationships.^7^,^8^ Although there is a significant volume of literature dedicated to the functionality of patients with BD, to the best of our knowledge, no studies have investigated the functional capacity of their caregivers.^9^-^11^

Considering the significant impact that caring for an individual with BD has on family members and caregivers, it would follow that both the functionality and QoL of this population are impaired. Therefore, the objectives of this study were: 1) to determine functionality levels in caregivers of individuals with BD, 2) to determine QoL levels in these individuals, and 3) to determine whether there is an association between functionality and QoL, as well as to investigate the possible relationship of these factors with sociodemographic and clinical characteristics (including the investigation of mental [MD] and personality disorders [PD]).

## Methods

### Participants and procedures

This cross-sectional study was conducted between 2020 and 2022 at the Centro de Estudos de Transtornos de Humor e Ansiedade, an outpatient psychiatry clinic at the Universidade Federal da Bahia’s Hospital Universitário Professor Edgard Santos, which is affiliated with the Empresa Brasileira de Serviços Hospitalares in Salvador, Bahia, Brazil.

A consecutive sample of caregivers was selected based on the following inclusion criteria: being the caregiver of a patient with BD or schizoaffective disorder, age ≥ 18 years, identified by the patient as the primary caregiver, and agreeing to participate in the study. We considered the primary caregiver to be the person the patient identified as providing the greatest emotional and financial support, most frequently accompanying the patient and responding in emergencies. These criteria are consistent with previous research on caregivers of individuals with mood disorders.^12^ Those who had difficulty appropriately responding to the instruments or who withdrew from the study were excluded. Of note, the individuals with schizoaffective disorder in this sample were due to diagnostic conversion from BD to schizoaffective disorder.

The caregiver interviews were preceded by confirming the patient’s BD diagnosis through a review of the hospital records or Centro de Estudos de Transtornos de Humor e Ansiedade data. Other patient data were collected, such as age, sex, self-reported race, occupation, BD subtype, and the current phase of the illness. Primary caregiver status was also confirmed by the attending physician, after which they were invited to participate in the study. If a caregiver met the inclusion criteria and both the patient and caregiver agreed, the researcher and participant proceeded to a suitable space for data collection. The participants were then briefed on the research objectives and procedures. After providing written informed consent, the caregiver then completed the research protocol. The assessments were conducted by two researchers (MT, SP) who had been previously trained to administer the instruments.

### Instruments

#### Sociodemographic and clinical questionnaire for caregivers

This questionnaire collected data on caregiver age, sex, education, marital status, occupation, income, kinship with the patient, whether the caregiver and patient resided together, and whether the caregiver had any non-psychiatric or psychiatric clinical comorbidities. The daily care provided for the patient was also assessed, including the duration of caregiver status, the frequency of outpatient clinic visits, whether the caregiver administered medications, etc.

#### World Health Organization Disability Assessment Schedule 2.0 (WHODAS 2.0)

This 12-item interviewer-administered instrument investigates functionality in the general population.^13^ The WHODAS 2.0 assesses health and disability, providing levels of functionality in six life domains: cognition, mobility, self-care, getting along, life activities, and participation (engaging in community and societal activities). Lower scores indicate better functionality.

#### The Short Form Health Survey (SF-36)^14^

This shortened version of the Medical Outcome Study consists of 36 items that represent eight dimensions of QoL: physical functioning, role-physical problems, bodily pain, general health, vitality, social functioning, role-emotional problems, and mental health. Scores for each dimension range from 0 to 100, with lower scores indicating lower QoL. A previous study considered the SF-36 appropriate for assessing outcomes among caregivers of individuals with mental illness.^15^

#### The Mini International Neuropsychiatric Interview

This structured interview investigates the main Axis I MD of the DSM IV and ICD-10.^16^

#### The Structured Clinical Interview for DSM-IV Axis II Personality Disorders

This structured clinical interview explores PD described in the DSM-IV.^17^

All questionnaires used in the research protocol were previously translated and validated for the Brazilian population.

### Statistical analysis

Categorical variables are presented as frequencies and were assessed using Fisher’s exact test or the chi-square test. The Shapiro-Wilk test was used to assess the normality of continuous variables, with analysis involving the *t*-test and the Mann-Whitney *U* test. Depending on the results of the Shapiro-Wilk test, the Pearson or Spearman correlation coefficient was applied to determine the correlation between WHODAS 2.0 and SF-36 results. The association between WHODAS 2.0 and SF-36 scores and clinical-demographic variables was evaluated through multiple linear regression. Individual raw WHODAS 2.0 scores were associated with percentiles defined by age parameters, as described by Andrews et al.^18^ in the general population. Scores exceeding the 75th percentile were considered indicative of functional impairment.

The model’s adjustment variables were chosen a priori: age, sex, degree of kinship, paid employment, physical activity, residence with the patient, duration of patient care, non-psychiatric illness, MD, and PD. The significance level was set at p ≤ 0.05. Missing data were excluded from the analysis. All analyses were performed in R 4.2.2.

### Ethics statement

This study followed the guidelines of the 1989 Helsinki Declaration and Brazilian National Health Council Resolution 466/2012 regarding research involving human subjects. The study was approved by the Hospital Universitário Professor Edgard Santos Research Ethics Committee (decision 4,389,592; 11/10/2020).

## Results

A total of 154 patients were selected to identify their respective caregivers, of whom 23 reported not having a caregiver. Seventeen caregivers were excluded due to incomplete data and eight others declined to participate in the study, thus the final sample consisted of 106 caregivers. Of these, the majority were women (69.8%), married or in a stable relationship (58.5%), and Black or of mixed race (81.1%); their median age was 52 (39-61) years. Regarding kinship, 22.7% were parents, 25.5% were children, 23.6% were siblings, 24.5% were spouses/partners, and 67% lived with the patient. MD, PD, or non-psychiatric clinical conditions occurred in 60.4%, 31.1%, and 59.4% of the sample, respectively (Table 1).

The majority of caregivers accompanied the patient to the outpatient clinic on appointment days (43.4% every time and 35.8% often). Additionally, 67% had been accompanying the patient for more than 10 years, 56.6% administered medications to the patient, 46.2% monitored the patient’s hygiene, 48.1% assisted with the patient’s diet, and 65.1% accompanied the patient in external activities (Table 1).

The patients’ median age was 55 (41-62) years, 75.5% were women, 42.4% were single, and 44.9% had completed high school. Most were Black or mixed race (84.2%) and were unemployed (80%). Regarding diagnosis, 81.1% were classified with type I BD, 12.3% with type II BD, and 6.6% with schizoaffective disorder. Concerning the mental state examination, 85.8% were in euthymia, 12.3% in mania/hypomania, and 1.9% in a depressive episode.

The median overall WHODAS 2.0 score was 17 (13-23). The median scores in SF-36 dimensions were 80 (55-95) for physical functioning, 75 (0-100) for role-physical problems, 62 (41-74) for bodily pain, 70 (51-85) for general health, 68 (45-80) for vitality, 88 (62-100) for social functioning, 100 (8-100) for role-emotional problems, and 76 (56-88) for mental health. The median WHODAS 2.0 score was negatively correlated with all eight domains of the SF-36 (p < 0.001) (Table 2).

Considering functional impairment as > 75th percentile, functionality was divided into two groups: those without functional impairment (n=51; 48.1%) and those with functional impairment (n=55; 51.9%). The median WHODAS 2.0 score in the non-impaired group was 13 (12.14), while that of the impaired group was 22 (20.26). According to the Pearson correlation, the impaired group’s higher mean score (lower functionality) was correlated with lower means in all SF-36 domains (worse QoL). The impaired group had more MDs (n=43; 78.2%), more PDs (n=24; 43.6%), and more non-psychiatric clinical diseases (n=40; 72.7%) than the non-impaired group (Table 3). No significant differences were found between the groups regarding sociodemographic variables.

In multiple linear regression, being the patient’s parent (5.65; p = 0.007), having a non-psychiatric clinical disease (4.09; p = 0.014) and having a MD (2.96; p = 0.043) were associated with lower functionality. Regarding QoL scores, being a parent (physical functioning [-28.41, p < 0.001], role-physical problems [-30.77, p = 0.017], bodily pain [-23.31, p = 0.001], general health [-19.58, p = 0.003], vitality [-26.89, p < 0.001], social functioning [-20.04, p = 0.004], role-emotional problems [-26.93, p = 0.030]), and having a non-psychiatric clinical disease (physical functioning [-21.24, p < 0.001], role-physical problems [-33.70, p = 0.001], bodily pain [-20.86, p < 0.001], general health [-14.43, p = 0.006], vitality [-15.62, p = 0.002], and mental health [-11.66, p = 0.013]) had the greatest association with lower QoL. Being female was negatively associated with role-physical problems (-11.66; p = 0.013), vitality (-13.02; p = 0.005), and mental health (-11.37; p = 0.010); MD was negatively associated with physical functioning (-11.55; p = 0.014), role-emotional problems (-31.18; p < 0.001), and mental health (-9.64; p = 0.020); and PD was negatively associated with vitality (-8.92; p = 0.042) and mental health (-8.36; p = 0.044). Advanced age was associated with better QoL in most domains (role-physical problems [1.25, p = 0.006], bodily pain [0.51, p = 0.035], general health [0.60, p = 0.009], vitality [0.57, p = 0.010], social functioning [0.68, p = 0.005], and mental health [0.52, p = 0.012]) (Table 4).

## Discussion

The aim of this study was to investigate the functionality and QoL levels in caregivers of patients with BD, considering that impairments in these areas can interfere with their physical and mental health, which may also negatively impact the care they provide. Clarifying these aspects and their association with caregiver clinical and sociodemographic factors was an innovation that could contribute to a deeper understanding of this population.

The results suggest that only about 50% of caregivers of individuals with BD exhibit functional impairment. The median WHODAS 2.0 score in those with impairment was 22 (interquartile range = 20.26), which is low considering that total scores can range from 12 to 60, with lower scores indicating higher functionality. Regarding caregiver QoL, surprisingly and unlike previous findings,^5^,^6^,^19^ we found high mean scores in the SF-36 domains, which suggests little impairment in QoL. These positive functionality and QoL results, which contradict our initial hypothesis, can be interpreted from two non-exclusive perspectives: the first is related to the patients, and the second is related to the sociodemographic and clinical characteristics of the caregivers themselves.

From the patients’ perspective, it can be inferred that this sample has better BD outcomes, given that the majority are regularly monitored and receive adequate medical and psychotherapeutic support. This context allows for better disease control with longer periods of remission, reducing the impact on caregivers. In fact, the results show that almost 90% of the patients were in symptom remission at the time of data collection.

From the caregiver’s perspective, it is assumed that the long-term patient follow-up (67% for > 10 years) in a specialized treatment center, which provides psychoeducation sessions for both parties, may have helped the caregivers develop coping strategies to better manage the patient’s disorder and better control their own mental health. Moreover, the possibility of selection bias should be noted due to variable caregiver adherence, i.e., that those who remained in the study might be more committed to the health care of the patient and themselves. However, regardless of explanatory factors for the satisfactory functionality and QoL levels of the sample, this study reveals that the caregiving process can be experienced without major distress, ensuring a good relationship between the patient and caregiver.

Positive aspects of caregiving, a construct used to assess positive caregiving experiences, may also explain these findings, even though they were not directly measured in this study. Previous studies involving caregivers of individuals with other chronic illnesses found a connection between developing skills and effectiveness in caring for sick family members and experiencing good emotions, such as feelings of accomplishment and gratification.^20^,^21^ Similarly, another study involving caregivers of individuals with BD found that better QoL in caregivers was associated with a more positive caregiving experience.^22^

The sociodemographic characteristics of our sample of caregivers align with another Brazilian study, especially regarding the preponderance of female caregivers and consanguineous relationships (i.e., parents, children, and siblings).^5^ International studies have reported similar findings in caregivers of individuals with other MD.^23^-^25^ The increased involvement of women and close family members, particularly mothers, may be influenced by societal norms, such as the cultural expectation that mothers should continue caring for their children even after they become adults. Although women were the majority in our sample, sex was not associated with functional level.

Of note, few functionality assessments of caregivers for patients with MD based on specific instruments (e.g., WHODAS 2.0) can be found in the literature, and we could find none on caregivers of individuals with BD, which prevents comparison of our results. To the best of our knowledge, the only study that has applied the WHODAS 2.0 to caregivers involved children who survived COVID-19.^26^

However, our finding that 59.4% of the sample had a non-psychiatric clinical condition may explain the limited impairment in both functionality and QoL, considering physical limitations, changes in sleep quality, social activity restrictions, and other symptoms resulting from illness. In fact, lower values were found only in the SF-36 bodily pain and vitality domains, and a strong correlation was found between WHODAS 2.0 results and non-psychiatric clinical conditions.

In general, considering the relationship between functionality and QoL, it can be said that the strong negative correlation between total WHODAS 2.0 scores and the domains of the SF-36, i.e. that greater functional impairment would be associated with lower QoL, was expected. Despite the scarcity of studies that have explored the direct relationship between these factors, lower QoL in caregivers is usually associated with various impairments, such as feelings of burden and depressive and anxious symptoms,^5^,^27^-^29^ which in turn can affect functionality. Given the above, we can assume that the results of our study are in line with the literature, i.e., that functionally impaired caregivers of individuals with BD have lower QoL scores, more non-psychiatric clinical diseases, more MD, and more PD.

We point out that the frequencies of MD (60.4%) and PD (31.1%) among these caregivers were higher than those of the global population (13%^30^ and 6.1%,^31^ respectively). Considering that 71.8% of these caregivers are consanguineous family members, the high rates may be due to susceptibility through genetic predisposition.^32^,^33^ Nevertheless, this vulnerability did not have a substantial impact on their functionality or QoL, as discussed above. Additionally, the high frequency of MD may have been influenced by the ongoing COVID-19 pandemic and its psychological and socioeconomic impact, leading to the development of various MDs in the general population, regardless of any caregiver role.^30^

In addition to the association between MD and lower levels of functionality, two other factors also influenced the variation in scores: being a parent and having a non-psychiatric illness. Parents of adult children with BD who have prior psychiatric symptoms are more vulnerable and are more likely to seek support in healthcare services.^34^ Thus, it is likely that the subsequent limitations of other clinical conditions, combined with the time spent caring for individuals with BD, could further weaken the health of these caregivers. A previous study found that caregivers of individuals with psychoses perceived worsening physical health over time, even without a significant change in QoL.^35^

In the present study, the factors associated with lower scores in at least one SF-36 domain were female sex, being a parent, non-psychiatric disease, MD, and PD. These results are consistent with a systematic review on factors associated with the QoL of relatives of adults with severe MD, including BD.^36^ Among the findings, the authors described worse QoL in women and parents, although the influence of the caregiver’s age on perceived QoL was inconsistent. Interestingly, in our study, older age was the only factor positively associated with six of the eight domains, i.e., the older the caregiver, the better the perceived QoL. Further investigation is needed to clarify this finding.

Finally, the negative association between QoL and MD is in line with previous findings that depressive and anxious symptoms are predictive of lower QoL.^5^,^29^,^37^ Moreover, PD was associated with the vitality and mental health domains of the SF-36. Indeed, symptoms such as emotional dysregulation, chronic stress, self-destructive behaviors, difficulty in interpersonal relationships, etc., which occur in MD and PD, can influence perceived energy levels and fatigue, as well as self-esteem, well-being, and other aspects of mental health.

In light of the above, investigating the level of functionality and QoL of caregivers of BD patients is of fundamental importance because social support can protect against worsening BD symptoms,^38^ which highlights the need to understand who the caregivers are, what their activities entail, and how they care for individuals with the disorder. Furthermore, interventions for caregivers have already been proposed, including a set of guidelines based on the daily needs reported by caregivers, patients, and physicians.^39^ Thus, through such guidelines and further in-depth investigations into social support, interventions can optimize comprehensive bidirectional care for patients and their caregivers.

This study has some limitations, the first of which is that, due to its cross-sectional design, causal relationships among the results could be established. The small sample size is another limitation, which was largely due to restrictions during the COVID-19 pandemic, when few patients and caregivers were treated in healthcare services. Furthermore, no comparisons were made with a control group of caregivers for individuals with different MDs.

However, the study’s strengths include its pioneering investigation of functionality using a specific scale in caregivers of individuals with BD, thus providing a basis for hypotheses and encouraging further research. It concurrently assessed functionality and QoL, shedding light on their association, which may affect patient care quality. Finally, another unprecedented aspect is the study’s investigation of MD and PD in caregivers through diagnostic interviews, examining their associations with functionality and QoL.

Functionality and QoL assessment in caregivers of BD patients showed that the greater the functional impairment, the lower the perceived QoL, which was influenced by high rates of MD and PD. However, the caregivers in this sample had satisfactory functionality and QoL, despite their high rates of MD and PD, in addition to the burden of caregiving. Finally, understanding these associations may be relevant because 1) they affect patients’ perceived social support and 2) they are useful for public health.

## Disclosure

The authors report no conflicts of interest.

## Figures and Tables

**Table 1:** Sociodemographic and clinical characteristics of caregivers of patients with bipolar disorder (n=106)

Characteristics	n (%)
Age (years)†	52 (39-61)
Women	74 (69.8)
Race	
Black	33 (31.1)
Mixed	53 (50.0)
White	20 (18.9)
Religious affiliation (yes)	90 (84.9)
Education	
≤ Primary school	27 (25.5)
High school	53 (50.0)
≥ University	26 (24.5)
Individual income (yes)	80 (75.5)
Individual income value (USD)†	220 (44-452)
Marital status	
Single	27 (25.5)
Married/steady partner	62 (58.5)
Separated/divorced	9 (8.5)
Widow/widower	8 (7.5)
Kinship with patient	
Parent	24 (22.7)
Sibling	25 (23.6)
Child	27 (25.5)
Spouse	26 (24.5)
Other	4 (3.8)
Lives with the patient (yes)	71 (67.0)
150 min/week physical activity (yes)	39 (36.8)
Non-psychiatric illness (yes)	63 (59.4)
Patient follow-up time (years)	
< 5	21 (19.8)
5-10	14 (13.2)
> 10	71 (67.0)
Mental disorder (years)‡	64 (60.4)
Personality disorder (yes)§ ^||^	32 (31.1)

Data presented as n (%), unless otherwise specified.

† Median (interquartile range).

‡ Mental disorders assessed using the Mini International Neuropsychiatric Interview Plus.

§ Personality disorders assessed using the Structured Clinical Interview for DSM-IV Axis II Personality Disorders.

|| Missing data (n=3).

**Table 2:** Correlation between functionality and quality of life in caregivers of patients with bipolar disorder (n=106)

SF- 36 domains	Score WHODAS 17 (13-23)	rho	p-value*
Physical functioning	80 (55-95)	-0.62	< 0.001
Role-physical problems	75 (0-100)	-0.59	< 0.001
Bodily pain	62 (41-74)	-0.68	< 0.001
General health	70 (51-85)	-0.56	< 0.001
Vitality	68 (45-80)	-0.61	< 0.001
Social functioning	88 (62-100)	-0.61	< 0.001
Role-emotional problems	100 (8-100)	-0.58	< 0.001
Mental health†	76 (56-88)	-0.65	< 0.001

BD = bipolar disorder; QoL = quality of life; SF-36 = 36-Item Short Form Health Survey; WHODAS 2.0 = World Health Organization Disability Assessment Schedule 2.0.

† Missing (n=1).

* p < 0.05.

**Table 3:** Frequencies and scores of caregivers of bipolar disorder patients with and without functional impairment (n=106)

Variables	Functional impairment†		
	Yes (n=55)	No (n=51)	p-value*
WHODAS (score)	22 (20-26)	13 (12-14)	< 0.001
SF-36 – Physical functioning	65 (40-88)	95 (80-100)	< 0.001
SF-36 – Role-physical problems	25 (0-75)	100 (88-100)	< 0.001
SF-36 – Bodily pain	51 (32-62)	74 (62-100)	< 0.001
SF-36 – General health	65 (45-75)	85 (65-92)	< 0.001
SF-36 – Vitality	50 (35-70)	80 (65-85)	< 0.001
SF-36 – Social functioning	75 (50-88)	100 (88-100)	< 0.001
SF-36 – Role-emotional problems	33 (0-100)	100 (100-100)	< 0.001
SF-36 – Mental health‡	60 (48-74)	84 (77-95)	< 0.001
Mental disorder (yes)	43 (78.2)	21 (41.2)	< 0.001
Personality disorder (yes)§	24 (43.6)	8 (15.7)	0.001
Non-psychiatric illness (yes)	40 (72.7)	23 (45.1)	0.004

Data presented as median (interquartile range) or n (%).

SF-36 = 36-Item Short Form Health Survey; WHODAS 2.0 = World Health Organization Disability Assessment Schedule 2.0.

† Classification based on age parameters according to Andrews et al.^18^

‡ Missing data (n=1).

§ Missing data (n=3).

* Fisher’s exact test; Wilcoxon rank sum test; Pearson’s chi-squared test; p < 0.05.

**Table 4:** Multiple linear regression for variables associated with functionality and QoL scores in caregivers of patients with BD

Variables	Coefficient and p-value of the WHODAS and by domain of the SF-36								
	WHODAS	PF	RPP	BP	GH	VT	SF	REP	MH
Age	-0.11 (p = 0.137)	0.36 (p = 0.120)	1.25 (p = 0.006)*	0.51 (p = 0.035)*	0.60 (p = 0.009)*	0.57 (p = 0.010)*	0.68 (p = 0.005)*	0.80 (p = 0.063)	0.52 (p = 0.012)*
Sex									
Male	-	-	-	-	-	-	-	-	-
Female	1.92 (p = 0.209)	-5.99 (p = 0.218)	-11.66 (p = 0.013)*	-9.06 (p = 0.074)	-2.48 (p = 0.605)	-13.02 (p = 0.005)*	-10.84 (p = 0.034)	-14.63 (p = 0.108)	-11.37 (p = 0.010)*
Kinship									
Sibling/child	-	-	-	-	-	-	-	-	-
Other	1.84 (p = 0.313)	-9.18 (p = 0.115)	-7.34 (p = 0.512)	-4.27 (p = 0.477)	-3.80 (p = 0.507)	-7.27 (p = 0.185)	0.15 (p = 0.980)	10.32 (p = 0.340)	-5.90 (p = 0.252)
Parent	5.65 (p = 0.007)*	-28.41 (p < 0.001)*	-30.77 (p = 0.017)*	-23.31 (p = 0.001)*	-19.58 (p = 0.003)*	-26.89 (p < 0.001)*	-20.04 (p = 0.004)*	-26.93 (p = 0.030)*	-11.36 (p = 0.054)
Paid employment									
No	-	-	-	-	-	-	-	-	-
Yes	0.29 (p = 0.858)	2.31 (p = 0.657)	-1.01 (p = 0.920)	-0.63 (p = 0.907)	2.26 (p = 0.660)	-7.20 (p = 0.145)	8.72 (p = 0.110)	9.17 (p = 0.345)	-0.03 (p = 0.994)
Physical activity (>150 min/week)									
No	-	-	-	-	-	-	-	-	-
Yes	-1.73 (p = 0.208)	8.23 (p = 0.061)	7.92 (p = 0.347)	2.79 (p = 0.536)	8.45 (p = 0.052)	7.66 (p = 0.065)	0.01 (p = 0.998)	-7.74 (p = 0.342)	0.42 (p = 0.913)
Non-psychiatric illness									
No	-	-	-	-	-	-	-	-	-
Yes	4.09 (p = 0.014)*	-21.24 (p < 0.001)*	-33.70 (p = 0.001)*	-20.86 (p < 0.001)*	-14.43 (p = 0.006)*	-15.62 (p = 0.002)*	-9.67 (p = 0.076)	-7.56 (p = 0.436)	-11.66 (p = 0.013)*
Resides with patient									
No	-	-	-	-	-	-	-	-	-
Yes	-1.06 (p = 0.530)	5.06 (p = 0.346)	-0.47 (p = 0.964)	3.25 (p = 0.559)	-3.92 (p = 0.460)	8.00 (p = 0.117)	5.62 (p = 0.315)	-6.75 (p = 0.500)	6.90 (p = 0.150)
Follow-up time (years)									
< 5	-	-	-	-	-	-	-	-	-
5-10	-0.03 (p = 0.991)	-3.42 (p = 0.653)	0.64 (p = 0.965)	-7.14 (p = 0.368)	3.22 (p = 0.670)	6.38 (p = 0.377)	8.02 (p = 0.314)	17.76 (p = 0.214)	1.70 (p = 0.802)
> 10	-0.34 (p = 0.850)	-5.64 (p = 0.325)	-7.51 (p = 0.497)	-8.76 (p = 0.142)	1.82 (p = 0.748)	0.47 (p = 0.930)	3.08 (p = 0.605)	0.82 (p = 0.938)	1.80 (p = 0.724)
Presence of MD									
No	-	-	-	-	-	-	-	-	-
Yes	2.96 (p = 0.043)*	-11.55 (p = 0.014)*	-11.40 (p = 0.202)	-6.19 (p = 0.197)	-5.54 (p = 0.226)	-4.12 (p = 0.345)	-9.68 (p = 0.046)	-31.18 (p < 0.001)*	-9.64 (p = 0.020)*
Presence of PD									
No	-	-	-	-	-	-	-	-	-
Yes	1.92 (p = 0.186)	0.36 (p = 0.937)	-4.26 (p = 0.631)	-8.24 (p = 0.085)	-3.80 (p = 0.403)	-8.92 (p = 0.042)*	-4.01 (p = 0.402)	0.36 (p = 0.967)	-8.36 (p = 0.044)*

BD = bipolar disorder; BP = bodily pain; GH = general health; MD = mental disorders; MH = mental health; PD = personality disorders; PF = physical functioning; QoL = quality of life; REP = role-emotional problems; RPP = role-physical problems; SF = social functioning; SF-36 = 36-Item Short Form Health Survey; VT = vitality; WHODAS 2.0 = World Health Organization Disability Assessment Schedule 2.0.

* p < 0.05.
